# A commonly used rumen-protected conjugated linoleic acid supplement marginally affects fatty acid distribution of body tissues and gene expression of mammary gland in heifers during early lactation

**DOI:** 10.1186/1476-511X-12-96

**Published:** 2013-07-04

**Authors:** Ronny Kramer, Simone Wolf, Tobias Petri, Dirk von Soosten, Sven Dänicke, Eva-Maria Weber, Ralf Zimmer, Juergen Rehage, Gerhard Jahreis

**Affiliations:** 1 Institute of Nutrition, Friedrich Schiller University Jena, Dornburger Str. 24, Jena, Germany; 2 Institute for Informatics, Ludwig-Maximilians-Universität, Munich, Germany; 3 Institute of Animal Nutrition, Friedrich-Loeffler-Institute (FLI), Federal Research Institute for Animal Health, Braunschweig, Germany; 4 Clinic for Cattle, University of Veterinary Medicine Hannover, Bischofsholer Damm 15, Hannover, Germany

**Keywords:** Conjugated linoleic acids, Milk fat depression, Fatty acid distribution, Liver, Mammary gland, Retroperitoneal fat, Gene expression

## Abstract

**Background:**

Conjugated linoleic acids (CLA) in general, and in particular the *trans-*10,*cis-*12 (*t*10,*c*12-CLA) isomer are potent modulators of milk fat synthesis in dairy cows. Studies in rodents, such as mice, have revealed that *t*10,*c*12-CLA is responsible for hepatic lipodystrophy and decreased adipose tissue with subsequent changes in the fatty acid distribution. The present study aimed to investigate the fatty acid distribution of lipids in several body tissues compared to their distribution in milk fat in early lactating cows in response to CLA treatment. Effects in mammary gland are further analyzed at gene expression level.

**Methods:**

Twenty-five Holstein heifers were fed a diet supplemented with (CLA groups) or without (CON groups) a rumen-protected CLA supplement that provided 6 g/d of *c*9,*t*11- and *t*10,*c*12-CLA. Five groups of randomly assigned cows were analyzed according to experimental design based on feeding and time of slaughter. Cows in the first group received no CLA supplement and were slaughtered one day postpartum (CON0). Milk samples were taken from the remaining cows in CON and CLA groups until slaughter at 42 (period 1) and 105 (period 2) days in milk (DIM). Immediately after slaughter, tissue samples from liver, retroperitoneal fat, mammary gland and *M*. *longissimus* (13th rib) were obtained and analyzed for fatty acid distribution. Relevant genes involved in lipid metabolism of the mammary gland were analyzed using a custom-made microarray platform.

**Results:**

Both supplemented CLA isomers increased significantly in milk fat. Furthermore, preformed fatty acids increased at the expense of *de novo-*synthesized fatty acids. Total and single *trans-*octadecenoic acids (e.g., *t*10-18:1 and *t*11-18:1) also significantly increased. Fatty acid distribution of the mammary gland showed similar changes to those in milk fat, due mainly to residual milk but without affecting gene expression. Liver fatty acids were not altered except for *trans-*octadecenoic acids, which were increased. Adipose tissue and *M*. *longissimus* were only marginally affected by CLA supplementation.

**Conclusions:**

Daily supplementation with CLA led to typical alterations usually observed in milk fat depression (reduction of *de novo*-synthesized fatty acids) but only marginally affected tissue lipids. Gene expression of the mammary gland was not influenced by CLA supplementation.

## Background

Conjugated linoleic acids (CLA) as products of partial hydrogenation of polyunsaturated fatty acids (PUFA) in ruminants [1], are attributed to have potent physiological effects. Among the numerous different positional and geometrical CLA isomers, *cis-*9,*trans-*11-18:2 (*c*9,*t*11-CLA) has quantitatively the highest proportion of dairy products whereas *t*10,*c*12-CLA is, besides *c*9,*t*11-CLA, mainly found in synthetically assembled CLA products. Each isomer has distinct chemical and biological properties, and may counteract one another when provided as a mixture. Distribution of isomers and total amount of CLA in ruminant products varies and depends on factors such as farm management [2] and feeding regimens [3]. Diets rich in PUFA such as for instance linoleic or linolenic acid and marine oils have been described to increase the amount of CLA in milk and tissue lipids and to bring about a reduction of fat content in milk of dairy cows [4,5]. This effect is termed low-fat milk syndrome or milk fat depression (MFD). In their review Bauman and Griinari [6] describe dietary induced MFD as being associated with a shift in the process of biohydrogenation leading to the formation of intermediates such as *t*10,*c*12-CLA and *t*10-18:1. Experiments with pure abomasally infused isomers in lactating cows identified *t*10,*c*12-CLA as the active isomer that decreases the concentration of milk fat [7] in a dose-dependent manner [8]. As a frequent byproduct of MFD, *t*10-18:1 is also attributed to reduce synthesis of milk fat in dairy cows [9,10], however Lock et al. [11] observed that pure abomasally infused *t*10-18:1 has no effect on milk fat synthesis.

The ability of *t*10,*c*12-CLA to induce MFD is a matter of interest, given that during early lactation nutritional requirements of dairy cows increase dramatically owing to the initiation of milk production [12]. The underlying mechanism by which *t*10,*c*12-CLA lowers the milk fat yield is due to reduced expression of lipogenic enzymes and transcription factors involved in lipid synthesis [13,14]. Hence, supplementation with rumen-protected CLA is an attempt to influence the energy output during the transition period and early lactation *via* milk fat. An improvement in the net energy balance and a reduction of body fat mobilization [15] diminish the incidence of metabolic disorders such as ketosis and milk fever and, in turn, the associated economic losses. In addition to its milk fat-lowering effect, *t*10,*c*12-CLA is also described as being capable of altering body composition by reducing adipose tissue and increasing the hepatic lipid content in different species [16-18]. These changes lead to alterations in the fatty acid distribution of liver and adipose tissue [19]. However, studies concerning these aspects have rarely been conducted in ruminants.

The objective of the present study was to analyze different tissue lipids from CLA-supplemented Holstein heifers in early lactation. Herein, we provide new data on CLA content and fatty acid distribution in liver, mammary gland, muscle and adipose tissue. With this new dataset a comparison between the effects of dietary CLA in rodents and ruminants is provided. The results of the fatty acid distribution in the mammary gland are supported by gene expression analysis. Thereby, the analysis of milk lipids provides reliable estimates on the extent of MFD and the enrichment of CLA after supplementation.

## Materials and methods

### Animals, diets and experimental design

Details of experimental design and feeding regimen have previously been published by von Soosten et al. [20]. In brief, twenty-five primiparous lactating German Holstein heifers were randomly assigned to 5 groups. All animals received a prepartum diet consisting of a partial mixed ration (PMR; 60% corn silage, 40% grass silage) *ad libitum* and additionally 2 kg of concentrate/d. An initial group of 5 cows (CON0) was slaughtered one day after parturition. Starting at 1 day postpartum, the remaining 20 heifers were fed a PMR (38% corn silage, 25% grass silage, 37% concentrate on dry matter basis) *ad libitum,* and additionally, 3.5 kg of concentrate/d which contained either 100 g of the CLA supplement (Lutrell® pure, BASF SE, Ludwigshafen, Germany) or 100 g of a control fat preparation (Silafat®, BASF SE). The CLA-supplemented diet provided a mixture of 6.0 g/d of *t*10,*c*12-CLA and 5.7 g/d of *c*9,*t*11-CLA isomer. At 42 days in milk (DIM; period 1), 5 cows from the control group (CON42) and 5 from the supplemented group (CLA42) were slaughtered. At 105 DIM (period 2), a further 5 cows from the control group (CON105) and 5 from the supplemented group (CLA105) were slaughtered. All animals had free access to water during the entire experimental trial. This animal trial was approved by the Lower Saxony State Office for Consumer Protection and Food Safety (LAVES, File No. 33.11.42502-04-071/07), Oldenburg, Germany.

### Sample collection

Milk samples were collected twice a week and stored at −20°C until freeze-dried. In addition, tissue samples from liver, mammary gland, *M*. *longissimus* (13th rib), and retroperitoneal adipose tissue were taken immediately after slaughter and stored at −20°C. Further, tissue samples for gene expression analysis of the mammary gland were also obtained and stored at −80°C.

### Analysis of lipids

Lipids from the collected milk samples were extracted in a Soxhlet apparatus using petroleum ether. The transesterification of milk lipids into fatty acid methyl esters (FAME) occurred as described by Kraft et al. [21].

Tissue samples were cut into small pieces and freeze-dried. Lipids were extracted from the freeze-dried tissue samples using a chloroform/methanol mixture according to the method of Folch et al. [22]. Conversion from liver and muscle tissue lipids into FAME was performed *via* acid-catalyzed transesterification using boron trifluoride (BF_3_) for 3 min upon prior ester cleavage with 0.5 M methanolic sodium hydroxyde for 10 min [23]. Since transesterification with BF_3_ is known to produce undesirable isomerization products of CLA [24], we reduced transesterification time from 5 to 3 min to minimize this problem. FAME were purified employing thin-layer chromatography (mobile phase: n-hexane/diethyl ether/glacial acetic acid; 85:15:0.2 by volume). Lipids from mammary gland and retroperitoneal adipose tissue were converted into FAME by means of base-catalyzed transesterification using 0.5 M sodium methoxide in anhydrous methanol.

Separation of the resulting FAME was undertaken *via* gas chromatography (GC-17A v.3, Shimadzu, Japan) and flame ionization detector (FID) in two steps. In the first step, FAME with a chain length of 4 to 26 carbon atoms were separated in a medium polar column (DB-225MS, 60 m × 0.25 mm i.d., 0.25 μm film thickness, Agilent Technologies, USA) following a temperature program [25]. In the second step, separation of *cis* and *trans* isomers of octadecenoic FAME was performed using a high-polarity column (Select™ FAME, 200 m × 0.25 mm i.d., 0.25 μm film thickness, Varian, Netherlands) under isothermal conditions at 176°C. Both separation steps possessed 260°C and 270°C temperature settings for injector and FID, respectively. Hydrogen was used as the carrier gas. According to the retention time of several standard mixtures, FAME were evaluated from all samples using an analytical GC software (GCsolution Version 2.31, Shimadzu, Japan).

To arrive at the final fatty acid distribution, a standard mixture consisting of *cis* and *trans* isomers of octadecenoic FAME was run on both columns. The total area of the *trans* isomers of octadecenoic FAME fatty acids on the medium polar column was repartitioned according to their individual peak areas on the high-polarity column using 18:0 from both columns as reference peak areas.

### Preparation of the microarray

Based on literature research and metabolic pathways, we selected 96 candidate genes (see Additional file 1) related to lipid and fatty acid metabolism (e.g., triacylglycerol synthesis, beta-oxidation, and desaturation of fatty acids) for the study. Gene expression analysis was performed employing a custom-made array platform integrated into a micro reaction tube (ArrayTube; Alere Technologies, Jena, Germany). For this purpose, total RNA from tissue samples (mammary gland) was extracted using the RNeasy® Plus Mini Kit (Qiagen, Hilden, Germany) according to the manufacturers’ instructions. Concentration and purity of the obtained RNA were assessed with the Nanodrop ND-1000 spectrophotometer (peQLab Biotechnology, Erlangen, Germany). To perform microarray-experiments, 1.5 μg RNA was transcribed to complementary DNA (cDNA) by simultaneously labeling with biotin-16-dUTP (Roche Diagnostics, Mannheim, Germany) using an expand reverse transcriptase (Roche Diagnostics), hexanucleotide random primer (Roche Diagnostics) and dNTPs (Fermentas, St. Leon-Rot, Germany). Hybridization was then undertaken *via* the kit obtained from Alere Technologies. In brief, purified and concentrated cDNA was hybridized to chip-bound oligonucleotides followed by conjugation with horseradish peroxidase-linked streptavidin. Subsequently, tetramethylbenzidine was added as substrate for the horseradish peroxidase to form precipitates. The precipitates were then recorded with a CCD-based transmission device (ATR03, Alere Technologies), and transferred to gray value data by means of a specialized array analysis software (IconoClust version 3.6, Alere Technologies).

### Analysis of expression data

Expression data was captured using the above described custom array platform for mammary gland. In total, we analyzed 62 chips with 111 probes each.

**Figure 1:**
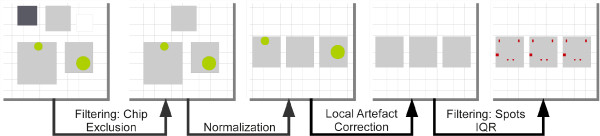
**Preprocessing workflow for expression analysis of custom array platform (Alere Technologies).** Each rectangle represents one chip, in which color intensity models measured signal intensities. Higher expression differences correspond to larger rectangles. Each circle represents physical distortion e.g. an air pocket. Red rectangles are spots that are excluded for further analysis.

We applied an error correction pipeline [26] to filter physical artefacts and defective chips were directly excluded. The most commonly used genes (ACTB, GAPDH) did not show a stable expression pattern across all chips. However, house-keeping genes or control sets, may suffer from unstable variance as previously described [27]. Thus, we relied on the frequently used locally-weighted regression scatterplot smoothing (LOWESS [28]) to normalize the set of arrays. Here, the *siggenes* package was used [29] as part of the R-project. We depended on case–control fold-changes with an associated moderated T-statistic (Significance of Microarrays, SAM, [29]) to quantify changes among feeding groups. The complete preprocessing workflow is shown in Figure 1.

Here, a two-step normalization strategy was applied. Firstly, an independent normalization for each of the five feeding groups was performed. Secondly, all sets of chips were normalized regardless of feeding group or time. Probes that did not exceed an inter-quartile-range threshold of 0.1 (IQR > 0.1) were filtered. Applying this cutoff, we excluded 47/94 out of 96 genes/207 probes. A probe is termed as significantly differentially expressed when its absolute fold-change exceeds 2 and its multiple-testing corrected significance threshold (q-value) is below 0.01.

Visual inspection was done using volcano-plots and log_2_(fold-changes) are scattered against their corresponding –log_10_(p-values) for each probe.

### Statistical analysis

Statistical analysis was performed using SPSS Statistics Version 19.0 (IBM, Armonk, USA). For the statistical analysis of fatty acids of milk samples, the linear mixed model of SPSS was applied. Hence, animals were considered as subjects and week of lactation as the repeated effect with an autoregressive covariance structure. Treatment, week of lactation, and the interaction between treatment and week of lactation were considered as factors for fixed effects. Animals were treated as random effects. Results are expressed as estimated marginal means (EMMeans) ± SEM. Statistical analysis of fatty acids of different tissues (liver tissue, retroperitoneal adipose tissue, muscle tissue, and tissue of the mammary gland) was performed by comparing the means of the CON groups with those of the CLA groups using the independent-samples *t*-test. Results are expressed as means ± SD. For all statistical analyses, observed differences are called significant if the calculated p-value is below 0.05.

## Results

### Milk samples

Supplementing the diet of dairy cows with equal amounts of *c*9,*t*11-CLA and *t*10,*c*12-CLA led to a significant increase of the two supplemented CLA isomers in milk lipids. In period 1, amounts of *c*9,*t*11-CLA and *t*10,*c*12-CLA increased from 3.5 to 4.4 g/day and from 0.01 to 0.42 g/day (Table 1), respectively. Similar increases were seen in period 2 (Table 2). Moreover, the proportion of other CLA isomers, mainly *c*9,*c*11-CLA and *t9*,*t*11-CLA also increased significantly in period 1. The rise in the two supplemented isomers was accompanied by a significant alteration of the fraction of saturated fatty acids (SFA) in both periods. SFA decreased from 708 to 585 g/day and from 781 to 608 g/day in period 1. In period 2, similar changes in SFA (Table 2) were observed. Due to some single *de novo-*synthesized fatty acids (DSFA) e.g. 6:0, 8:0 and 12:0, the total amount of DSFA was significantly reduced by treatment in both periods. In contrast, the total amount of preformed fatty acids (PRFA) did not change. In both periods, the sum of fatty acids with 16 carbon atoms was notably reduced, due to the significant decline of palmitic acid (16:0), the most abundant fatty acid in milk lipids. Except for significantly elevated levels of *t*12-18:1 and *c*12-18:1 in period 1, other MUFA were found increased but failed to reach significance. The fatty acid ratio *c*9-14:1/14:0 was significantly reduced in period 1 indicating inhibition of Δ9 desaturase activity.

**Table 1:** Selected milk lipid fatty acids during period 1 (1 until 42 DIM)

**Fatty acid [g/day]**	**Treatment**^**1**^		**SEM**	***P*-value**	
	**CON**	**CLA**		**Trt**	**Trt x WL**^**2**^
4:0	37.5	37.0	2.6	0.906	0.528
6:0	27.2	22.9	1.5	0.051	0.496
8:0	15.4	12.1*	0.74	0.005	0.809
10:0	36.9	26.8*	1.6	0.000	0.987
12:0	39.3	27.8*	1.7	0.000	0.926
14:0	11.5	92.4*	4.2	0.005	0.992
*c*9-14:1	6.3	4.5*	0.36	0.002	0.561
15:0	12.0	10.1*	0.59	0.035	0.991
16:0	301	237*	11.8	0.001	0.199
*c*9-16:1	12.2	12.3	0.84	0.924	0.436
17:0	6.1	6.0	0.35	0.728	0.658
18:0	97.8	92.1	8.4	0.633	0.402
*t*6/*t*7/*t*8-18:1	1.6	2.1	0.17	0.063	0.663
*t*9-18:1	2.0	2.3	0.12	0.088	0.414
*t*10-18:1	4.2	6.8	1.1	0.096	0.822
*t*11-18:1	7.3	8.7	0.77	0.215	0.240
*t*12-18:1	2.1	2.6*	0.15	0.030	0.352
*t*13/*t*14-18:1	2.3	2.6	0.16	0.227	0.993
*c*9-18:1	172	183	16.0	0.633	0.336
*c*11-18:1	8.1	8.5	0.63	0.602	0.649
*c*12-18:1	1.7	2.1*	0.14	0.032	0.605
*c*13-18:1	0.91	1.0	0.08	0.662	0.249
18:2 n-6	14	14.8	0.95	0.571	0.016
18:3 n-3	0.23	0.2	0.01	0.104	0.857
CLA					
*c*9,*t*11-CLA	3.5	4.4*	0.30	0.039	0.787
*t*10,*c*12-CLA	0.01	0.42*	0.02	0.000	0.125
Other CLA	0.74	0.97*	0.07	0.022	0.787
Summations					
∑ SFA	708	585*	29.5	0.008	0.617
∑ MUFA	230	246	18.9	0.563	0.394
∑ PUFA	25.3	27.8	1.8	0.335	0.143
∑ *trans*-18:1	24.3	30.7	2.18	0.054	0.998
∑ BCFA^3^	16.4	15.7	0.74	0.520	0.567
∑ C16^4^	314	249*	12.4	0.002	0.191
∑ *de novo*^5^	300	246*	11.7	0.003	0.950
∑ preformed^6^	350	365	28.2	0.716	0.262
Ratio					
*c*9-14:1/14:0	0.54	0.41*	0.03	0.006	0.434

^1^Treatment (Trt): cows in the control (CON) group (n = 10) received a control fat supplement, in which the CLA were substituted with stearic acid. Cows in the conjugated linoleic acid (CLA) group (n = 10) consumed 6 g/d each of *trans*-10,*cis*-12 CLA and *cis*-9,*trans*-11 CLA.

^2^Week of lactation; ^3^*BCFA* branched-chain fatty acids, ^4^*C16* Sum of 16:0 and *c*9-16:1.

^*5*^*de novo*: fatty acids <16 carbon atoms; ^6^preformed: fatty acids >16 carbon atoms.

*Estimated mariginal means (EMMeans) are defined as significant (P < 0.05).

**Table 2:** Selected milk lipid fatty acids during period 2 (>42 until 105 DIM)

**Fatty acid [g/day]**	**Treatment**^**1**^		**SEM**	***P*-value**	
	**CON**	**CLA**		**Trt**	**Trt x WL**^**2**^
4:0	37.3	33.5	1.9	0.186	0.470
6:0	29.8	23.3*	1.6	0.016	0.767
8:0	16.6	11.8*	1.2	0.011	0.824
10:0	41.1	27.5*	3.2	0.015	0.888
12:0	47.1	30.4*	3.8	0.013	0.931
14:0	129	104	8.1	0.059	0.858
*c*9-14:1	11.0	6x.9	1.3	0.052	0.977
15:0	14.8	10.2	1.5	0.055	0.430
16:0	364	267*	19.7	0.009	0.346
*c*9-16:1	15.2	12.8	1.3	0.240	0.800
17:0	5.8	5.0	0.4	0.182	0.410
18:0	72.3	72.9	4.5	0.927	0.261
*t*6/*t*7/*t*8-18:1	1.2	1.6	0.15	0.075	0.250
*t*9-18:1	1.7	2.1	0.14	0.078	0.484
*t*10-18:1	2.6	4.3	0.62	0.080	0.708
*t*11-18:1	5.0	6.1	0.38	0.073	0.364
*t*12-18:1	2.1	2.6	0.20	0.121	0.220
*t*13/*t*14-18:1	2.5	2.7	0.21	0.590	0.033
*c*9-18:1	139	150	8.5	0.375	0.388
*c*11-18:1	6.0	6.4	0.67	0.671	0.624
*c*12-18:1	1.9	2.4	0.19	0.075	0.253
*c*13-18:1	0.7	0.8	0.06	0.664	0.911
18:2 n-6	13.3	14.5	0.97	0.384	0.924
18:3 n-3	0.31	0.24	0.02	0.069	0.253
CLA					
* c*9,*t*11-CLA	3.4	4.3	0.34	0.093	0.767
* t*10*,c*12-CLA	0.00	0.37*	0.04	0.000	0.030
Other CLA	0.73	0.91	0.07	0.078	0.211
Summations					
∑ SFA	781	608*	39.9	0.014	0.582
∑ MUFA	199	208	11.5	0.626	0.444
∑ PUFA	24.5	26.8	1.7	0.374	0.762
∑ *trans*-18:1	19.7	24.7	1.9	0.103	0.254
∑ BCFA^3^	17.1	16.0	0.81	0.347	0.346
∑ C16^4^	380	280*	20.9	0.010	0.366
∑ *de novo*^5^	345	261*	21.4	0.021	0.947
∑ preformed^6^	282	301	13.9	0.358	0.291
Ratio					
*c*9-14:1/14:0	0.86	0.55	0.10	0.060	1.000

^1^Treatment (Trt): cows in the control (CON) group (n = 10) received a control fat supplement, in which the CLA were substituted with stearic acid. Cows in the conjugated linoleic acid (CLA) group (n = 10) consumed 6 g/d each of *trans*-10,*cis*-12 CLA and *cis*-9,*trans*-11 CLA.

^2^Week of lactation; ^*3*^*BCFA* branched-chain fatty acids; ^4^*C16* Sum of 16:0 and *c*9-16:1.

^*5*^*de novo*: fatty acids <16 carbon atoms; ^6^preformed: fatty acids >16 carbon atoms.

*Estimated mariginal means (EMMeans) are defined as significant (P < 0.05).

### Mammary gland

At 42 DIM and 105 DIM, the fatty acid profile of the mammary gland was characterized by elevated concentrations of the two supplemented isomers. The *t*10,*c*12-CLA isomer could not be detected in the CON groups but was present in the CLA groups (Table 3). Although, a decline in SFA and an increase in monounsaturated fatty acids (MUFA) were observed at 42 DIM and 105 DIM, the levels were insignificant. At 42 DIM, concentrations of both polyunsaturated fatty acids (PUFA) and total n-6 PUFA were significantly increased mediated through linoleic acid. At 105 DIM, PUFA and total n-6 PUFA remained unaffected. However, the total percentage of *t*-18:1 acids increased significantly at 42 DIM and fatty acid ratio remained unaffected (Table 3).

**Table 3:** Selected fatty acids in lipids of the mammary gland at 42 DIM and 105 DIM of supplementation

**Fatty acid [% FAME]**	**Period 1 (42 DIM)**		**Period 2 (105 DIM)**	
	**CON**	**CLA**	**CON**	**CLA**
	**Mean ± SD**	**Mean ± SD**	**Mean ± SD**	**Mean ± SD**
14:0	9.88 ± 0.70	9.40 ± 2.10	9.10 ± 0.87	9.13 ± 1.67
*c*9-14:1	0.65 ± 0.19	0.51 ± 0.19	0.83 ± 0.02	0.68 ± 0.27
16:0	32.62 ± 3.16	30.16 ± 4.28	31.41 ± 3.64	29.05 ± 3.13
*c*9-16:1	1.52 ± 0.16	1.49 ± 0.12	1.84 ± 0.10	1.89 ± 0.25
18:0	10.38 ± 1.43	11.84 ± 2.56	9.80 ± 2.00	11.17 ± 2.05
*t*6/*t*7/*t*8-18:1	0.08 ± 0.05	0.14 ± 0.02*	0.13 ± 0.02	0.17 ± 0.07
*t*9-18:1	0.20 ± 0.01	0.25 ± 0.02*	0.23 ± 0.01	0.28 ± 0.06
*t*10-18:1	0.22 ± 0.06	0.37 ± 0.09*	0.29 ± 0.05	0.68 ± 0.68
*t*11-18:1	0.65 ± 0.13	0.77 ± 0.04	0.68 ± 0.06	0.89 ± 0.37
*t*12-18:1	0.21 ± 0.03	0.29 ± 0.02*	0.27 ± 0.02	0.31 ± 0.05
*t*13/*t*14-18:1	ND	ND	ND	ND
*c*9-18:1	20.45 ± 2.88	22.46 ± 3.11	22.34 ± 2.56	22.82 ± 5.00
*c*11-18:1	0.88 ± 0.13	1.06 ± 0.11*	0.84 ± 0.17	1.12 ± 0.24
*c*12-18:1	0.22 ± 0.03	0.31 ± 0.01*	0.28 ± 0.02	0.33 ± 0.04*
*c*13-18:1	0.08 ± 0.02	0.10 ± 0.01	0.10 ± 0.02	0.11 ± 0.02
18:2 n-6	2.52 ± 0.40	3.43 ± 0.57*	3.08 ± 0.27	4.02 ± 1.23
18:3 n-3	0.42 ± 0.09	0.53 ± 0.09	0.41 ± 0.03	0.49 ± 0.10
20:4 n-6	0.37 ± 0.08	0.46 ± 0.11	0.48 ± 0.06	0.65 ± 0.14
20:5 n-3	0.13 ± 0.03	0.17 ± 0.05	0.12 ± 0.01	0.16 ± 0.03*
22:5 n-3	0.25 ± 0.05	0.32 ± 0.10	0.27 ± 0.03	0.33 ± 0.07
22:6 n-3	0.03 ± 0.01	0.03 ± 0.01	0.02 ± 0.01	0.03 ± 0.01
CLA				
*c*9,*t*11-CLA	0.37 ± 0.03	0.49 ± 0.11	0.51 ± 0.06	0.73 ± 0.34
*t*10*,c*12-CLA	ND	0.05 ± 0.01	ND	0.05 ± 0.01
Other CLA	0.06 ± 0.02	0.09 ± 0.01*	0.07 ± 0.02	0.10 ± 0.04
Summations				
∑ SFA	69.04 ± 3.20	64.84 ± 3.74	65.13 ± 2.65	62.13 ± 5.51
∑ MUFA	26.33 ± 2.89	29.02 ± 2.90	29.12 ± 2.76	30.59 ± 4.73
∑ PUFA n-3	0.91 ± 0.14	1.17 ± 0.25	0.93 ± 0.05	1.12 ± 0.18
∑ PUFA n-6	3.29 ± 0.55	4.34 ± 0.71*	4.23 ± 0.33	5.27 ± 1.41
∑ PUFA incl. CLA	4.64 ± 0.69	6.14 ± 0.86*	5.74 ± 0.39	7.28 ± 1.94
∑ *trans*-18:1	1.81 ± 0.34	2.37 ± 0.20*	2.11 ± 0.15	2.87 ± 1.25
Ratio				
*c*9-14:1/14:0	0.07 ± 0.02	0.05 ± 0.01	0.09 ± 0.01	0.07 ± 0.02

Cows in the control (CON) group (n = 5) of each period received a control fat supplement, in which the CLA were substituted with stearic acid. Cows in the conjugated linoleic acid (CLA) group (n = 5) of each period consumed 6 g/d each of *trans*-10,*cis*-12 CLA and *cis*-9,*trans*-11 CLA.

*Means **±** SD for each period are defined significant (*P* < 0.05).

*ND* not detected.

### Liver tissue

In liver tissue, *t*10,*c*12-CLA was not detected in both periods 1 and 2 although the proportion of *c*9,*t*11-CLA tended to increase. *c*12-18:1, as well as linoleic acid (18:2 n-6) were significantly elevated in both periods (Table 4). In addition, proportions of *t*-18:1 acids, *t*10-18:1 and docosahexenoic acid (DHA; 22:6 n-3) were found increased at 42 DIM but fatty acid ratio was not affected (Table 4).

**Table 4:** Selected fatty acids in liver lipids at 42 DIM and 105 DIM of supplementation

**Fatty acid [% FAME]**	**Period 1 (42 DIM)**		**Period 2 (105 DIM)**	
	**CON**	**CLA**	**CON**	**CLA**
	**Mean ± SD**	**Mean ± SD**	**Mean ± SD**	**Mean ± SD**
14:0	1.18 ± 0.69	1.46 ± 0.43	0.69 ± 0.24	0.67 ± 0.17
*c*9-14:1	0.19 ± 0.13	0.18 ± 0.14	0.16 ± 0.11	0.07 ± 0.08
16:0	14.37 ± 4.59	13.95 ± 1.92	10.93 ± 1.54	11.42 ± 3.75
*c*9-16:1	1.35 ± 0.77	1.36 ± 0.46	0.76 ± 0.31	0.65 ± 0.31
18:0	28.41 ± 4.93	28.44 ± 2.35	32.34 ± 1.16	31.47 ± 4.48
*t*6/*t*7/*t*8-18:1	0.03 ± 0.02	0.05 ± 0.01	0.04 ± 0.01	0.05 ± 0.03
*t*9-18:1	0.13 ± 0.02	0.16 ± 0.02	0.15 ± 0.03	0.16 ± 0.07
*t*10-18:1	0.08 ± 0.02	0.15 ± 0.02*	0.08 ± 0.03	0.30 ± 0.42
*t*11-18:1	0.56 ± 0.11	0.74 ± 0.13	0.49 ± 0.08	0.80 ± 0.52
*t*12-18:1	0.22 ± 0.08	0.31 ± 0.04	0.24 ± 0.04	0.27 ± 0.03
*t*13/*t*14-18:1	0.60 ± 0.44	0.50 ± 0.14	0.79 ± 0.05	0.62 ± 0.38
*c*9-18:1	15.31 ± 4.49	14.21 ± 2.28	11.96 ± 0.81	12.10 ± 5.46
*c*11-18:1	1.79 ± 0.10	1.92 ± 0.12	1.56 ± 0.15	1.60 ± 0.30
*c*12-18:1	0.18 ± 0.02	0.29 ± 0.05*	0.18 ± 0.04	0.27 ± 0.07*
*c*13-18:1	0.15 ± 0.01	0.17 ± 0.05	0.19 ± 0.04	0.16 ± 0.06
18:2 n-6	9.04 ± 0.67	9.93 ± 0.25*	9.06 ± 1.17	11.13 ± 1.12*
18:3 n-3	0.83 ± 0.30	0.99 ± 0.27	0.58 ± 0.09	0.79 ± 0.23
20:4 n-6	5.43 ± 0.75	5.37 ± 1.05	6.10 ± 0.72	6.02 ± 0.58
20:5 n-3	1.50 ± 0.47	1.55 ± 0.21	1.24 ± 0.13	1.29 ± 0.43
22:5 n-3	3.70 ± 0.81	3.76 ± 0.58	4.12 ± 0.44	4.12 ± 0.97
22:6 n-3	0.62 ± 0.09	0.79 ± 0.11*	0.43 ± 0.07	0.53 ± 0.14
CLA				
*c*9,*t*11-CLA	0.24 ± 0.10	0.29 ± 0.07	0.19 ± 0.06	0.26 ± 0.13
*t*10*,c*12-CLA	ND	ND	ND	ND
Other CLA	0.14 ± 0.10	0.12 ± 0.05	0.16 ± 0.02	0.10 ± 0.06
Summations				
∑ SFA	48.95 ±1.11	49.08 ± 0.57	49.08 ± 0.97	48.62 ± 1.75
∑ MUFA	22.03 ± 5.11	21.58 ± 3.03	17.99 ± 1.21	18.57 ± 5.26
∑ PUFA n-3	7.71 ± 1.41	8.34 ± 0.48	7.68 ± 0.66	7.84 ± 1.58
∑ PUFA n-6	20.92 ± 3.74	20.59 ± 2.30	24.90 ± 1.26	24.60 ± 2.77
∑ PUFA incl. CLA	29.02 ± 4.96	29.34 ± 2.50	32.93 ± 1.79	32.81 ± 3.89
∑ *trans*-18:1	2.10 ± 0.43	2.51 ± 0.17	2.42 ± 0.17	2.95 ± 1.33
Ratio				
*c*9-14:1/14:0	0.18 ± 0.12	0.11 ± 0.06	0.21 ± 0.11	0.11 ± 0.10

Cows in the control (CON) group (n = 5) of each period received a control fat supplement, in which the CLA were substituted with stearic acid. Cows in the conjugated linoleic acid (CLA) group (n = 5) of each period consumed 6 g/d each of *trans*-10,*cis*-12 CLA and *cis*-9,*trans*-11 CLA.

*Means ± SD for each period are defined significant (*P* < 0.05).

*ND* not detected.

### *M*. *longissimus*

As with retroperitoneal adipose tissue, supplementation of CLA isomers had no significant effect on the proportion of *c*9,*t*11-CLA, and *t*10,*c*12-CLA in both periods (Table 5). The concentration of SFA increased whereas the levels of MUFA considerably decreased in period 2 which is due to a significant decline of the most abundant oleic acid (*c*9-18:1) in muscle tissue. With the exception of *c*9-14:1/14:0, ratios of other fatty acids were not significantly affected by CLA supplementation.

**Table 5:** **Selected fatty acids in lipids of *M*. *longissimus *at 42 DIM and 105 DIM of supplementation**

**Fatty acid [% FAME]**	**Period 1 (42 DIM)**		**Period 2 (105 DIM)**	
	**CON**	**CLA**	**CON**	**CLA**
	**Mean ± SD**	**Mean ± SD**	**Mean ± SD**	**Mean ± SD**
14:0	1.73 ± 0.37	1.79 ± 0.28	1.74 ± 0.31	1.67 ± 0.52
*c*9-14:1	0.49 ± 0.20	0.41 ± 0.05	0.57 ± 0.20	0.36 ± 0.25
16:0	25.42 ± 2.39	25.38 ± 1.52	25.55 ± 1.38	25.09 ± 2.32
*c*9-16:1	3.74 ± 1.01	3.75 ± 0.48	4.11 ± 0.80	3.61 ± 1.15
18:0	13.54 ± 1.31	13.38 ± 0.82	12.07 ± 1.26	13.59 ± 1.68
*t*6/*t*7/*t*8-18:1	0.12 ± 0.02	0.12 ± 0.02	0.14 ± 0.01	0.13 ± 0.02
*t*9-18:1	0.30 ± 0.02	0.31 ± 0.04	0.32 ± 0.02	0.32 ± 0.03
*t*10-18:1	0.24 ± 0.02	0.26 ± 0.01	0.29 ± 0.04	0.36 ± 0.07
*t*11-18:1	0.52 ± 0.05	0.54 ± 0.09	0.52 ± 0.05	0.53 ± 0.10
*t*12-18:1	0.13 ± 0.02	0.13 ± 0.01	0.14 ± 0.01	0.15 ± 0.02
*t*13/*t*14-18:1	ND	ND	ND	ND
*c*9-18:1	43.01 ± 1.76	40.94 ± 3.22	43.41 ± 1.38	41.04 ± 1.76*
*c*11-18:1	1.81 ± 0.22	1.90 ± 0.19	2.09 ± 0.34	1.78 ± 0.17
*c*12-18:1	0.12 ± 0.05	0.12 ± 0.04	0.13 ± 0.03	0.14 ± 0.05
*c*13-18:1	0.27 ± 0.05	0.20 ± 0.02	0.30 ± 0.08	0.18 ± 0.08*
18:2 n-6	2.29 ± 0.52	3.18 ± 1.36	2.53 ± 1.04	3.22 ± 1.14
18:3 n-3	0.36 ± 0.09	0.52 ± 0.17	0.34 ± 0.08	0.43 ± 0.12
20:4 n-6	0.66 ± 0.18	1.04 ± 0.69	0.69 ± 0.38	1.15 ± 0.74
20:5 n-3	0.14 ± 0.05	0.29 ± 0.23	0.14 ± 0.10	0.26 ± 0.18
22:5 n-3	0.25 ± 0.06	0.42 ± 0.23	0.24 ± 0.16	0.43 ± 0.24
22:6 n-3	ND	0.03 ± 0.03	ND	0.02 ± 0.02
CLA				
*c*9,*t*11-CLA	0.19 ± 0.03	0.19 ± 0.04	0.19 ± 0.03	0.21 ± 0.03
*t*10*,c*12-CLA	ND	ND	ND	ND
Other CLA	0.07 ± 0.01	0.07 ± 0.02	0.08 ± 0.01	0.08 ± 0.02
Summations				
∑ SFA	44.32 ± 1.21	44.58 ± 1.57	42.86 ± 0.98	44.48 ± 0.75*
∑ MUFA	51.30 ± 1.09	49.11 ± 2.99	52.42 ± 2.18	49.08 ± 2.11*
∑ PUFA n-3	0.82 ± 0.18	1.35 ± 0.69	0.79 ± 0.36	1.22 ± 0.60
∑ PUFA n-6	3.30 ± 0.71	4.69 ± 2.27	3.65 ± 1.51	4.91 ± 2.15
∑ PUFA incl. CLA	4.38 ± 0.91	6.31 ± 2.95	4.71 ± 1.82	6.44 ± 2.76
∑ *trans*-18:1	1.46 ± 0.13	1.46 ± 0.12	1.49 ± 0.04	1.60 ± 0.22
Ratio				
*c*9-14:1/14:0	0.27 ± 0.06	0.23 ± 0.03	0.32 ± 0.07	0.20 ± 0.07*

Cows in the control (CON) group (n = 5) of each period received a control fat supplement, in which the CLA were substituted with stearic acid. Cows in the conjugated linoleic acid (CLA) group (n = 5) of each period consumed 6 g/d each of *trans*-10,*cis*-12 CLA and *cis*-9,*trans*-11 CLA.

*Means ± SD for each period are defined significant (*P* < 0.05).

*ND* not detected.

### Retroperitoneal tissue

At 42 DIM and 105 DIM, the fatty acid profile of retroperitoneal adipose tissue showed no significant changes with regard to the proportion of *c*9,*t*11-CLA. However, the proportion of the *t*10,*c*12-CLA isomer changed from non-detectable to 0.02% of FAME only at 105 DIM. Furthermore, a significant elevation of n-3 PUFA from 0.20 to 0.25% of FAME was observed at 105 DIM. Other fatty acid parameters remained unaffected (see Additional file 2).

### Expression data

**Figure 2:**
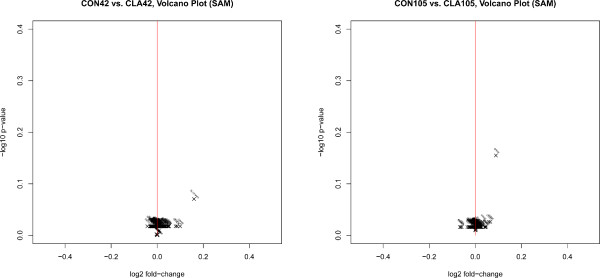
**Results of expression analysis on probe level for the 2 feeding groups (CON and CLA) in period 1 (left) and period 2 (right) are shown as volcano-plots.** A probe is called significant if its absolute fold-change is above 2 (|log2(fold-change)| > 1) and the corresponding p-value is below 0.01 (–log10(p-Value) > 2).

The above mentioned effects observed with FAME were analyzed on gene expression level in mammary gland where the highest changes in fatty acid composition apart from milk have been observed. Results of the probe-level SAM analysis between the 2 feeding groups in periods 1 and 2 are shown in Figure 2.

None of the compared periods yielded results sufficient to draw final conclusions with respect to alterations at expression levels caused by feeding the CLA mixture. In addition to the described periods, further group combinations over feeding and time were also inspected. The strongest (insignificant) changes were observed for different time-points and feeding groups (e.g. CON0 - CLA105). Based on this data, CLA supplementation could thus affect speed rather than the amount of metabolites participating in the underlying processes. Another explanation for the insignificant results could be due to other confounding factors which affect and obscure the changes. A possible confounding factor could be the health status of the animal at day of measurement.

## Discussion

In the present study, both measurement periods were characterized by elevated concentrations of the supplemented isomers in milk lipids following supplementation with 5.7 g/d *c*9,*t*11-CLA and 6 g/d *t*10,*c*12-CLA (Tables 1 and 2). According to Baumgard et al. [7], elevated concentrations of *t*10,*c*12-CLA are linked to reductions in milk fat content and/or milk fat yield in a dose-dependent manner. We have previously shown that the given dose of CLA was potent towards reducing the content of milk fat significantly by 14.1% in period 1 and by 25.4% in period 2. Further, CLA led to a decrease in the milk fat yield by 17.1% in period 2 in early lactation [20]. This is in accordance with data from the study by Moallem et al. [30], in which 4.7 g/d *t*10,*c*12-CLA and 4.7 g/d *c*9,*t*11-CLA of the same lipid-encapsulated supplement was used and showed similar reductions in milk fat content (13%) and milk fat yield (9%, insignificant) compared to period 1 of the present study. Further, our results are also supported by observations from Giesy et al. [31], who reported a reduction in milk fat content of approximately 14% by supplementing four CLA isomers as a mixture that, amongst other CLA isomers, contained 4.4 g/d or 8.6 g/d of *t*10,*c*12-CLA. Although the main effect on the milk fat-lowering effect is ascribed to *t*10,*c*12-CLA, a possible participation or interaction of the other isomers (e.g. *c*9,*t*11-CLA, *c*8,*t*10-CLA or *c*11,*t*13-CLA) cannot be excluded.

The reduction in milk fat content and milk fat yield in the present study is marked by a significant decrease in single DSFA (8:0, 10:0 and 12:0) as well as 16:0. The latter fatty acid originated to about 50% each from *de novo-*synthesis and from circulating fatty acids. Castaneda-Gutierrez et al. [32] showed similar results on single DSFA, ΣC16, and fatty acid ratios with the exception that the latter were unaffected when provided with 9.2 g/d *t*10,*c*12-CLA as part of a rumen-protected CLA-mix supplement as Ca-salts. Interestingly, Bernal-Santos et al. [33] used a CLA-mix consisting of four CLA isomers (*c*9,*t*11-CLA; *t*10,*c*12-CLA; *t*8,*c*10-CLA and *c*11,*t*13-CLA) that provided 8.8 g/d *t*10,*c*12-CLA as Ca-salts, and reported no significant effect on DSFA, except for ΣC16 fatty acids. In the study [33], the fatty acid ratios also remained unaffected. As proposed by Baumgard et al. [34] and Peterson et al. [8], low doses of *t*10,*c*12-CLA show no effect on Δ9-desaturase activity despite significant reduction of milk fat content and/or milk fat yield. In contrast, abomasal infusions of several doses of *t*10,*c*12-CLA resulted in alterations of fatty acid ratios indicating inhibition of Δ9-desaturase activity [7,14,34,35]. The *c*9-14:1/14:0 ratio provides a suitable estimation of Δ9 desaturase activity that is specific for the mammary gland and thus for milk lipids, given that 14:0 and *c*9-14:1 are almost exclusively derived by *de novo*-synthesis in the mammary gland [36]. In the present study, we could show a significant reduction of the *c*9-14:1/14:0 ratio in period 1, indicating inhibition of Δ9 desaturase activity. This is in accordance with our observations on DSFA and ΣC16 in the present study. In addition, because the amount of *c*12-18:1 was significantly elevated in period 1, it might have further enhanced the inhibition of Δ9 desaturase activity [37].

MFD, either diet induced or mediated by *t*10,*c*12-CLA, is caused by down-regulation of several transcription factors [13,38] and enzymes involved in *de novo*-synthesis, desaturation, and elongation [14,39] processes. Based on our gene expression analysis, we did not observe a treatment-related effect on genes coding for relevant enzymes or transcription factors in lipid metabolism in the mammary gland that could explain the reduction of the *c*9-14:1/14:0 ratio.

*T*-18:1 acids, intermediates of partial hydrogenation of unsaturated fatty acids, especially of linoleic acid are often linked to diets that cause MFD [40]. Several research groups using rumen-protected CLA, observed a significant increase of single 18:1 fatty acids (e.g., *t*9-, *t*10-, *t*11-18:1) in milk fat [32,33,41]. The results of our study concur with these findings as we observed similar elevations in single *t*-18:1 acids which were close to significance in both periods (Tables 1 and 2).

Increased concentrations of *t*-18:1 acids, especially *t*10-18:1 and *t*11-18:1, suggest a poor protection rate of the supplemented CLA. Investigating the duodenal availability of the same lipid-encapsulated CLA supplement, Pappritz et al. [42] showed protection rates of 16% and 5% after providing 3 and 8 g/d of *t*10,*c*12-CLA, respectively, and assumed an impaired rumen protection due to the pelleting process. Because we used the same conditions for preparing of the CLA supplement, it is most likely that unprotected CLA serve as potential substrates for microbial alterations and, consequently, lead to elevated concentrations of *t*-18:1 acids. Since Shingfield et al. [10] recently showed that a mixture of *t*-18:1 acids, in particular *t*10-18:1 and *t*11-18:1, are capable of inducing MFD, it is most likely that the reduction of milk fat yield and milk fat content in the present study is caused by both *t*10,*c*12-CLA and, to a lesser extent by *t*10-18:1.

CLA, originally identified in fried ground beef, gained considerable attention because of its anticarcinogenic effects [43]. Other studies with CLA supplemented in feed either as a mixture or as pure isomers showed specific changes in body composition that are almost exclusively due to the *t*10,*c*12-CLA isomer [44,45]. Jahreis et al. [46] reported in their summary that liver and adipose tissue, and to a certain extent muscle tissue are most affected by CLA. However, these changes are not consistent among species. The majority of studies investigating the effects of CLA on body composition and/or fatty acid distribution have mainly been conducted in rodents and pigs. The few studies in dairy cattle, concentrated on the mammary gland as the principal object of research.

In the present study, supplementation with CLA showed only marginal effects on the fatty acid distribution of the above mentioned tissues. Despite supplementing a mixture with equal amounts of both isomers, *t*10,*c*12-CLA was detected only in the mammary gland (Table 3) and retroperitoneal adipose tissue (Additional file 2), and not in the lipids of liver and *M*. *longissimus*. Kramer et al. [47] showed that the distribution of the different CLA isomers incorporated into liver and heart lipids in pigs is by no means equal for all lipid classes but rather specific for each individual CLA isomer. Furthermore, Kramer et al. [47] showed that the percentage of *t*10,*c*12-CLA is generally low in liver lipids compared to *c*9,*t*11-CLA. This observation has been previously reported [19,48] and is explained as a preferable conversion of *t*10,*c*12-CLA through elongation and desaturation or elevated beta-oxidation in liver and muscle which finally results in lower concentrations than the supplementation might predict [44,49]. Further, we have to concede that emerging isomerization products due to transesterification with BF_3_ may have reduced the already low concentration of *t*10,*c*12-CLA.

Mice fed diets with different amounts of CLA, in particular pure *t*10,*c*12-CLA or in combination with *c*9,*t*11-CLA, exhibited decreased concentrations of linoleic acid and increased accumulation of oleic acid in liver lipids which, in turn, led to significantly higher liver weights [16,19,50,51]. This is in contrast to the results of the present study, where liver weights increased in a fashion typical during early lactation but independent of CLA supplementation [20]. Furthermore, changes of the fatty acid distribution in liver lipids were limited to linoleic acid and *c*12-18:1 in both periods, and to *t*10-18:1 and DHA in period 1 (Table 4). Increased percentages of linoleic acid and DHA in liver lipids in the present study could be explained by a shift in the triglyceride:phospholipid ratio because phospholipids contain higher portions of long-chain fatty acids [52]. Based on an average dry matter intake of 15.3 kg in both periods, the dose of the two supplemented CLA isomers at 0.04% is low in the present study compared to approximately 0.5% – 1% used in rodent or pig models [53]. *C*9,*t*11- and *t*10,*c*12-CLA, administered as pure isomers are described to have opposite effects on body composition and fatty acid distribution in mice. For example, Kelley et al. [51] showed a decrease of oleic acid in total lipids of liver, retroperitoneal adipose tissue, and spleen after administration of 0.5% *c*9,*t*11-CLA, whereas 0.5% *t*10,*c*12-CLA increased oleic acid in liver and heart lipids, and decreased linoleic and arachidonic acid in liver lipids.

Studies in rodents showed that the retroperitoneal adipose tissue was most sensitive to CLA [54-56]. Although we observed a significant reduction of the retroperitoneal adipose tissue as part of the empty body weight in dairy cattle [20], this effect is not necessarily linked to a changed fatty acid distribution as the present study demonstrated.

## Conclusion

In the present study, we showed that the daily supplemented dose of 5.7 g/d *c*9,*t*11-CLA and 6.0 g/d *t*10,*c*12-CLA predominantly affected the mammary gland and, thus milk lipids by decreasing DSFA and C16, leading to MFD. The significantly reduced *c*9-14:1/14:0 ratio and considerably increased *c*12-18:1 isomer underline these observations. However, except for elevated concentrations of certain single fatty acids in liver and muscle tissue, the effects on fatty acid distribution in different body tissues remained marginal at this dose of CLA and could be the result of a slight shift in the triglyceride:phospholipid ratio.

## Abbreviations

BCFA: Branched-chain fatty acids; BF3: Boron trifluoride; cDNA: Complementary DNA; CLA: Conjugated linoleic acids; CON: Control group; DIM: Days in milk; DSFA: *de novo*-synthesized fatty acids; EMMeans: Estimated marginal means; FAME: Fatty acid methyl esters; FID: Flame ionization detector; IQR: Inter-quartile-range; LOWESS: Locally-weighted regression scatterplot smoothing; MFD: Milk fat depression; MUFA: Monounsaturated fatty acids; PMR: Partial mixed ration; PRFA: Preformed fatty acids; PUFA: Polyunsaturated fatty acids; SAM: Significance of microarrays; SFA: Saturated fatty acids

## Competing interests

The authors declare that they have no competing interests.

## Authors’ contributions

RK participated in tissue sample collection, performed fatty acid analysis *via* gas chromatography, gene expression using a custom-made micro array, did statistical evaluation of fatty acid data and wrote the manuscript. DS carried out the animal study. SW designed and implemented the analysis pipeline of the provided expression data, interpreted expression data and results, drafted the expression analysis as part of the manuscript and reviewed the complete manuscript critically. TP designed and implemented the analysis pipeline of the provided expression data, reviewed the expression analysis draft and the complete manuscript critically. SD participated in study design and reviewed the manuscript. EW helped in sample collection. RZ helped in analysis of expression data and reviewed the manuscript. JR participated in study design. GJ participated in study design, helped drafting the manuscript. All authors read and approved the final manuscript.

## Supplementary Material

Additional file 1 Candidate genes and their corresponding chip-bound probe sequences. Click here for file

Additional file 2 Selected fatty acids in lipids of retroperitoneal adipose tissue at 42 DIM and 105 DIM of supplementation. Click here for file
