# Supplementary material for: A commonly used rumen-protected conjugated linoleic acid supplement marginally affects fatty acid distribution of body tissues and gene expression of mammary gland in heifers during early lactation
Source: Lipids Health Dis. 2013 Jul 4;12:96. doi: 10.1186/1476-511X-12-96 (PMC3706325; doi:10.1186/1476-511X-12-96)
Supplement: Additional file 1 — Candidate genes and their corresponding chip-bound probe sequences. [file 1476-511X-12-96-S1.pdf]

Additional file 1 Candidate genes and their corresponding chip-bound probe sequences.

No.	Symbol	Name	Sequence
1	ACAA1	acetyl-CoA acyltransferase 1	CCATCCCTGTGGCTTTGCAGAAAGCAGG
2	ACAA2	acetyl-CoA acyltransferase 2	GGATGTGATCCCACTATCATGGGTATTGGTCCC
3	ACACA	acetyl-CoA carboxylase alpha	TGTCCTTCTCCTCCAACCTCAACCACTACG
4	ACACB	acetyl-CoA carboxylase beta	TGCCCACTTTCTTCTACCACGCTAACAAAGTG
5	ACADL	acyl-CoA dehydrogenase, long chain	TGTCTCCAGCTGCACAGAGTGAAACGTCT
6	ACADM	acyl-CoA dehydrogenase, C-4 to C-12 straight chain	GGAGAATGACTGAGGAGCCATTAATGTGTGCCT
7	ACADS	acyl-CoA dehydrogenase, C-2 to C-3 short chain	GGTCTTCGCCAGCACCGACAGATCC
8	ACADSB	acyl-CoA dehydrogenase, short/branched chain	GAGGGCCTTCATGTAGGGAAACCAGAGAACA
9	ACADVL	acyl-CoA dehydrogenase, very long chain	AGATGGGCATCAAAGCCTCAAACACAGCAG
10	ACSL1	acyl-CoA synthetase long-chain family member 1	AGCGGCATCATCAGAAACAACAGCCTGTG
11	ACSL4	acyl-CoA synthetase long-chain family member 4	TGAGAGAATTCCTGGGCTGGGACCAAAGG
12	ACSS1	acyl-CoA synthetase short-chain family member 1	CATCTTCGGCTGCGTGGCTGATATCGG
13	ACSS2	acyl-CoA synthetase short-chain family member 2	CCTAGCCAATCTCCTCCGATTAAGAGGCCATG
14	ACTB	actin, beta	TCCGTGACATCAAGGAGAAGCTCTGCTACG
15	ADIPOR1	adiponectin receptor 1	CCACCACGCCATGGAGAAGATGGAGGA
16	ADIPOR2	adiponectin receptor 2	GCCATTCTCTGCCTTTCTTTCTCATGGCTGT

No.	Symbol	Name	Sequence
17	AGPAT1	1-acylglycerol-3-phosphate O-acyltransferase 1	GATGCCATCAGTGTCATGTCTGAGGTCGC
18	AGPAT2	1-acylglycerol-3-phosphate O-acyltransferase 2	AGGCCCAGGTACCCATCATCCCTGT
19	AGPAT3	1-acylglycerol-3-phosphate O-acyltransferase 3	CCGAATACATGTGGTTCCTCCTGTACTGTGAGG
20	AGPAT4	1-acylglycerol-3-phosphate O-acyltransferase 4	CTGATTCACTGCGAGGGCACACGGT
21	AGPAT5	1-acylglycerol-3-phosphate O-acyltransferase 5	TCTGTGGATGATAAAGGGCAGCGGAAGGA
22	AGPAT6	1-acylglycerol-3-phosphate O-acyltransferase 6	TGGAACAGCAGCAAGTACGGGATGGTG
23	CEBPA	CCAAT/enhancer binding protein (C/EBP), alpha	GCAACGTGGAGACGCAGCTGAAGGT
24	CEBPB	CCAAT/enhancer binding protein (C/EBP), beta	GGTCAAGAGCAAGGCCAAGAAGACGGTG
25	CEBPD	CCAAT/enhancer binding protein (C/EBP), delta	TGGAGCTGTGCCACGATGAGCTCTTC
26	CEBPE	CCAAT/enhancer binding protein (C/EBP), epsilon	CGCATCCTGGAGACACAGCAGAAGGTG
27	CEBPG	CCAAT/enhancer binding protein (C/EBP), gamma	CCCATGGATCGCAACAGTGACGAGTACC
28	CEBPZ	CCAAT/enhancer binding protein (C/EBP), zeta	AGCCTCTTCTACCTGGATGAAGGCGATTGT
29	CPT1A	carnitine palmitoyltransferase 1B-like	CTCCACAGCAGGTGGAACTGTTCAATTTGGA
30	CPT1B	carnitine palmitoyltransferase 1B (muscle)	GCGACTCCAGTGGGACATTCCTAAGCAG
31	CPT2	carnitine palmitoyltransferase 2	CGGTTTCAGAGAGGAGGCAGAGAATTCCTGAAG
32	DCI	dodecenoyl-CoA isomerase	TGAACGAGACCCTGCTGGGCATCATC
33	DGAT1	diacylglycerol O-acyltransferase 1	TCTTCTACTGGCTCTTCCACTCCTGCCTGA

No.	Symbol	Name	Sequence
34	DGAT2	diacylglycerol O-acyltransferase 2	CGGGAGTACCTGATGTCTGGAGGCATCT
35	ECH1	enoyl CoA hydratase 1, peroxisomal	TCTTCCCAGACAAGGAGAGCATGCTTGATGC
36	ECHS1	enoyl CoA hydratase, short chain, 1, mitochondrial	CACTGGGACCAGCTCACACGTGTCAAG
37	EHHADH	enoyl-CoA, hydratase/3-hydroxyacyl CoA dehydrogenase	TGCAAAGGCCCAGATCCCTGTGATTGC
38	ELOVL1	elongation of very long chain fatty acids (FEN1/Elo2, SUR4/Elo3, yeast)-like 1	TCTCCGGAAGAAAGACGGACAGGTGACC
39	ELOVL2	elongation of very long chain fatty acids (FEN1/Elo2, SUR4/Elo3, yeast)-like 2	ACGTCTACCACCACGCCTCCATGTTTAACA
40	ELOVL3	elongation of very long chain fatty acids (FEN1/Elo2, SUR4/Elo3, yeast)-like 3	CCTTCCTGTTTGTTCTCAGCAAGGTCGTTGA
41	ELOVL4	elongation of very long chain fatty acids (FEN1/Elo2, SUR4/Elo3, yeast)-like 4	TGTTGCAGCTGGTTCAGTTCCACGTGAC
42	ELOVL5	ELOVL family member 5, elongation of long chain fatty acids (FEN1/Elo2, SUR4/Elo3-like, yeast)	CCAGATCACGGTCCTGCATGTGTATCACCA
43	ELOVL6	ELOVL family member 6, elongation of long chain fatty acids (FEN1/Elo2, SUR4/Elo3-like, yeast)	ACTGGTACCACCACATCACTGTGCTTCTGT
44	ELOVL7	ELOVL family member 7, elongation of long chain fatty acids (yeast)	TGCCATGGACCTGGTGGTTTGGAGTCA
45	FABP1	fatty acid binding protein 1, liver	AGATCAAGGCAGTGGTTCAGCAGGAAGGT
46	FABP2	fatty acid binding protein 2, intestinal	TCCGAGAAATTATAGGTGGCGAGATGGTCCAGA
47	FABP3	fatty acid binding protein 3, muscle and heart (mammary-derived growth inhibitor)	GCACCTTCAAGAACACAGAGATCAGCTTCAAGC
48	FABP4	fatty acid binding protein 4, adipocyte	ACTCCAGATGACAGGAAAGTCAAGAGCATCGT
49	FADS1	fatty acid desaturase 1	CGCTGTTGGGACTGAAAGGCTTCCTGG
50	FADS2	fatty acid desaturase 2	GTGAACATGCTGCACGTGTTTGTCCTGG

No.	Symbol	Name	Sequence
51	FASN	fatty acid synthase	CTGCACACGCTGCTCAGAGGACACC
52	FFAR2	free fatty acid receptor 2	AGAATGAGACCACCTGCTACGAGAACTTCACC
53	FFAR3	free fatty acid receptor 3	GGCTAGAGATGGCAGTGGTCCTCTTTGGG
54	GAPDH	glyceraldehyde-3-phosphate dehydrogenase	TCGTGGAGGGACTTATGACCACTGTCCAC
55	GPAM	glycerol-3-phosphate acyltransferase, mitochondrial	CCAGCTATACTTCCCTCAAGACCCAGTGGTG
56	HADH	hydroxyacyl-CoA dehydrogenase	CAGCAACACTTCCTCTTTGCAGATCACAAGCC
57	HP	haptoglobin	CTCATTGGATGCCAAGGGCAGCTTTCCC
58	IL6	interleukin 6 (interferon, beta 2)	TCCAATCTGGGTTCAATCAGGCGATTTGCTTG
59	INSIG1	insulin induced gene 1	TCCTGGCCTTGGTGCTCAACCTCCT
60	INSIG2	insulin induced gene 2	TGGCCTTGGAGTAGGAATTGCTTTCTTGGC
61	LEP	leptin	ACCAGTCTGCCTTCCAGAAATGTGGTCCA
62	LEPR	leptin receptor	CCTATGATGCAGTGTACTGCTGCAATGAGCA
63	LIPE	lipase, hormone-sensitive	GTCCTCGTCAGGCTCATCTCCTATGACCTG
64	LPIN1	lipin 1	CCATGCAGGCCTTCCAGAAACCATTGC
65	LPIN2	lipin 2	ACCTGGCCACCTCACCAATCCCTACT
66	LPIN3	lipin 3	CCTCCGCCTCTCCTCCAGTCAGATCC
67	LPL	lipoprotein lipase	GGATCAGCTGGTGAAGTGCTCCCACG

No.	Symbol	Name	Sequence
68	MCAT	malonyl CoA:ACP acyltransferase (mitochondrial)	GAGGCCATGCAGGAAGCCTCGGAAG
69	MLX	MAX-like protein X	AGCACACACTCAGGTCGAGCAGAAGAGG
70	MLXIPL	MLX interacting protein-like	CACACACCAGCGGTTCGACCAAATGC
71	NR1H2	nuclear receptor subfamily 1, group H, member 2	CAACCACGAGACTGAGTGCATCACCTTCC
72	NR1H3	nuclear receptor subfamily 1, group H, member 3	AGACGTCTGCGATTGAGGTGATGCTCCT
73	PLA2G2D1	calcium-dependent phospholipase A2 PLA2G2D1	ACCCAGAGATGCCACAGACAGGTGCT
74	PLA2G2D3	calcium-dependent phospholipase A2 PLA2G2D3	ACAAGACGTTGGCCTTCTGCCTGCA
75	PLA2G2D4	calcium-dependent phospholipase A2 PLA2G2D4	CAACCCAGAGATGCCACAGACAGGTGC
76	PLA2G2D5	calcium-dependent phospholipase A2 PLA2G2D5	ACAAGACGTTGGCCTTCTGCCTGCAG
77	PLA2G4A	phospholipase A2, group IVA (cytosolic, calcium-dependent)	TGTGGTGGCCATACTGGGCTCAGGT
78	PLCB1	phospholipase C, beta 1 (phosphoinositide-specific)	TCCAAAGGGAACACGCGTGGATTCATCTAAC
79	PLCB3	phospholipase C, beta 3 (phosphatidylinositol-specific)	GTCCACACTCGTCAACTATATCGAGCCTGTCA
80	POR	P450 (cytochrome) oxidoreductase	CAAGATGGCCTCCTCGTCAGGCGAG
81	PPARA	peroxisome proliferator-activated receptor alpha	TGGACTTGAATGACCAAGTCACTCTGCTGAAGT
82	PPARD	peroxisome proliferator-activated receptor delta	ACCTGAGGGCCTTCTCCAAGCACATCT
83	PPARG	peroxisome proliferator-activated receptor gamma	CGACCAACTGAACCCAGAGTCTGCTGAC
84	PTGS1	prostaglandin-endoperoxide synthase 1	CCACGATATGGCTGCGTGAGCACAAC

No.	Symbol	Name	Sequence
85	PTGS2	prostaglandin-endoperoxide synthase 2	TGGTGCCTGGTCTGATGATGTATGCCAC
86	SCAP	SREBF chaperone	CTCTGGAGGAAACTGTCCTTCCGCCACT
87	SCD	stearoyl-CoA desaturase (delta-9-desaturase)	CGAACCTACAAAGCTCGGCTGCCTCT
88	SCD5	stearoyl-CoA desaturase 5	CTGACCCTGTGGTCCGGTTCCAGAGA
89	SLC25A20	solute carrier family 25 (carnitine/acylcarnitine translocase), member 20	GTGATGATCCGAGCCTTCCCAGCCA
90	SLC27A6	solute carrier family 27 (fatty acid transporter), member 6	TCTGGAGTGACCTCTGGTACCTGCTGAAGA
91	SLC2A4	solute carrier family 2 (facilitated glucose transporter), member 4	AGAGAAACCAGCCTATGCCACCATTGGAG
92	SREBF1	sterol regulatory element binding transcription factor 1	CTGGTCTGGCTGATGAATGGGCTGCT
93	SREBF2	sterol regulatory element binding transcription factor 2	CTGGATGATGCCAACGCTCCTCCTGT
94	THRSP	thyroid hormone responsive	ACAACTACTTCACCATGCTCAAGGCCATCC
95	TNF	tumor necrosis factor	CCAACGGTGTGAAGCTGGAAGACAACCAG
96	UCP1	uncoupling protein 1 (mitochondrial, proton carrier)	TCAGACTGCAAGCTCAGAGCCATCTCCAC

Candidate genes for the custom array platform (ArrayTube; Alere Technologies, Jena, Germany) and their corresponding chip-bound oligonucleotide sequence.
