# Supplementary material for: A commonly used rumen-protected conjugated linoleic acid supplement marginally affects fatty acid distribution of body tissues and gene expression of mammary gland in heifers during early lactation
Source: Lipids Health Dis. 2013 Jul 4;12:96. doi: 10.1186/1476-511X-12-96 (PMC3706325; doi:10.1186/1476-511X-12-96)
Supplement: Additional file 2 — Selected fatty acids in lipids of retroperitoneal adipose tissue at 42 DIM and 105 DIM of supplementation. [file 1476-511X-12-96-S2.pdf]

Additional file 2 Selected fatty acids in lipids of retroperitoneal adipose tissue at 42 DIM and 105 DIM of supplementation.

	Period 1 (42 DIM)		Period 2 (105 DIM)		
	CON	CLA	CON	CLA	
Fatty acid [% FAME]	Mean ± SD	Mean ± SD	Mean ± SD	Mean ± SD	
14:0	2.13 ± 0.29	2.29 ± 0.31	2.41 ± 0.32	2.59 ± 0.53	
*c*9-14:1	0.14 ± 0.02	0.14 ± 0.04	0.23 ± 0.07	0.22 ± 0.08	
16:0	22.30 ± 2.26	22.69 ± 1.20	23.11 ± 1.59	24.35 ± 2.20	
*c*9-16:1	0.90 ± 0.19	0.95 ± 0.13	1.30 ± 0.40	1.59 ± 0.30	
18:0	35.35 ± 2.51	34.46 ± 1.31	31.15 ± 4.47	29.32 ± 3.13	
*t*6/*t*7/*t*8-18:1	0.17 ± 0.02	0.15 ± 0.02	0.14 ± 0.02	0.15 ± 0.04	
*t*9-18:1	0.20 ± 0.01	0.19 ± 0.03	0.18 ± 0.02	0.22 ± 0.03	
*t*10-18:1	0.19 ± 0.02	0.20 ± 0.03	0.20 ± 0.05	0.36 ± 0.20	
*t*11-18:1	1.00 ± 0.15	1.06 ± 0.20	0.91 ± 0.17	0.91 ± 0.22	
*t*12-18:1	0.22 ± 0.03	0.21 ± 0.03	0.19 ± 0.03	0.22 ± 0.05	
*t*13/*t*14-18:1	0.24 ± 0.04	0.21 ± 0.06	0.20 ± 0.03	0.23 ± 0.06	
*c*9-18:1	28.51 ± 3.00	28.90 ± 2.26	32.02 ± 3.61	31.02 ± 3.63	
*c*11-18:1	0.54 ± 0.05	0.58 ± 0.07	0.64 ± 0.10	0.72 ± 0.10	
*c*12-18:1	0.09 ± 0.03	0.11 ± 0.02	0.11 ± 0.02	0.14 ± 0.04	
*c*13-18:1	0.05 ± 0.01	0.05 ± 0.02	0.07 ± 0.03	0.06 ± 0.02	
18:2 n-6	0.58 ± 0.08	0.61 ± 0.06	0.65 ± 0.09	0.82 ± 0.16	
18:3 n-3	0.20 ± 0.09	0.20 ± 0.06	0.15 ± 0.03	0.20 ± 0.04	
20:4 n-6	ND	ND	ND	ND	
20:5 n-3	ND	ND	ND	ND	
22:5 n-3	0.03 ± 0.01	0.05 ± 0.01	ND	0.03 ± 0.01	
22:6 n-3	ND	ND	ND	ND	
CLA					
*c*9,*t*11-CLA	0.18 ± 0.02	0.19 ± 0.03	0.18 ± 0.03	0.23 ± 0.05	
*t*10,*c*12-CLA	ND	ND	ND	0.02 ± 0.01	
Other CLA	0.13 ± 0.04	0.13 ± 0.04	0.11 ± 0.03	0.10 ± 0.01	
Summations					
∑ SFA	65.21 ± 2.99	64.67 ± 2.07	61.45 ± 4.21	61.44 ± 3.86	
∑ MUFA	33.44 ± 3.02	33.91 ± 2.11	37.23 ± 4.12	36.96 ± 3.81	
∑ PUFA n-3	0.27 ± 0.10	0.29 ± 0.07	0.20 ± 0.03	0.25 ± 0.04	*
∑ PUFA n-6	0.77 ± 0.10	0.80 ± 0.06	0.83 ± 0.08	1.00 ± 0.20	
∑ PUFA incl. CLA	1.35 ± 0.23	1.42 ± 0.16	1.32 ± 0.10	1.60 ± 0.29	
∑ *trans*-18:1	2.60 ± 0.28	2.57 ± 0.43	2.28 ± 0.26	2.60 ± 0.44	
Ratios					
*c*9-14:1/14:0	0.07 ± 0.01	0.06 ± 0.01	0.10 ± 0.03	0.08 ± 0.02	

Cows in the control (CON) group (n=5) of each period received a control fat supplement, in which the CLA were substituted with stearic acid. Cows in the conjugated linoleic acid (CLA) group (n=5) of each period consumed 6 g/d each of *trans*-10,*cis*-12 CLA and *cis*-9,*trans*-11 CLA.

*Means ± SD for each period are defined significant ($P < 0.05$)

ND = not detected
